# Back in My Day: A Journal Club Using Landmark Articles for Emergency Medicine-Bound Medical Students

**DOI:** 10.5811/westjem.2019.12.44527

**Published:** 2019-12-19

**Authors:** Christopher E. San Miguel, Cynthia Leung, Nicholas E. Kman, Jason Bischof

**Affiliations:** The Ohio State University Wexner Medical Center, Department of Emergency Medicine, Columbus, Ohio

## Abstract

This journal club style curriculum was developed to advance 4th year medical students in Emergency Medicine (EM) Milestone 19. The curriculum was introduced as part of a longitudinal boot camp course for EM- bound students. Students met monthly with faculty members to critically evaluate landmark articles within the field of EM. The curriculum culminated with student group presentations of two contemporary research articles with opposing conclusions. Discussed articles covered the following topics: stroke care, head trauma, cervical spine trauma, pulmonary embolism, cardiology treatments, syncope, post- cardiac arrest care, pediatrics, sepsis, and fluid resuscitation. The curriculum was evaluated using the institution’s standard student educational session evaluation form. Students rated the quality of the sessions highly, and based on thematic review of comments, the journal club was a beneficial addition to the boot camp curriculum.

## BACKGROUND

The ability to critically appraise scholarly literature and apply results to patient care is a core component of medical practice as evidenced by its inclusion as a milestone for emergency medicine (EM) trainees by the Accreditation Council for Graduate Medical Education (ACGME).^1^ Milestones are the knowledge, skills and attitudes required for successful practice within a given specialty, organized in a developmental framework from levels 1 through 5 to demonstrate advancing proficiency. Level 1 milestones are those skills expected on the first day of residency. The level 1 anchor for EM Milestone 19, “Practice-based Performance Improvement” requires that a learner “describes basic principles of evidence-based medicine,” whereas one of the level 3 anchors of the same milestone requires that a learner “demonstrates the ability to critically appraise scientific literature and apply evidence-based medicine to improve one’s individual performance.”^2^

Traditionally, medical schools have focused their curricula on teaching principals of evidence-based medicine (EBM) during the first two years of medical school, and EM residency programs have focused on teaching clinical practice application via journal clubs.^3^,^4^ While medical schools report formal EBM activities occurring in the clinical environment during the third year of medical school, the most common environment for fourth- year EBM training was in fact “none.”^3^ Consequently, it is possible that new EM trainees could arrive at residency having not used or revisited EBM concepts at all over the final year of their medical school training and having never been exposed to EBM application within the field of EM.

Our institution has a longitudinal boot camp course for medical students pursuing an EM residency.^5^ This boot camp experience was redesigned after the introduction of the ACGME Milestone Project in an effort to teach and assess EM-bound medical students on their progress toward and beyond level 1 proficiency.^6^ In an effort to better teach and assess EM Milestone 19, a curriculum was designed to reinforce core concepts of EBM while exposing senior medical students to historical articles that had widespread practice-changing effects.

## OBJECTIVES

The culminating objective of our curriculum was to enable students to analyze a recently published article and discuss its implications in clinical practice, thus ensuring that each student at minimum had achieved level 1 in EM Milestone 19. To gain the skills necessary to complete this objective the students 1) analyzed landmark articles in EM to become familiar with common research methodologies, 2) studied the development process of clinical decision rules and the limitations of their application to clinical practice, and 3) discussed how research findings have historically influenced practice change within EM.

## CURRICULAR DESIGN

This EBM curriculum was delivered in a traditional journal club format. Sessions were held monthly throughout the academic year and ran on average 90 minutes. Two faculty members, one fellowship-trained in education and the other fellowship-trained in research, co-facilitated all but two of the sessions. Both faculty members had experience facilitating small group discussions, but no further specific training was undertaken prior to the implementation of the curriculum. There were 10 total sessions, and to accommodate away rotations and interviews, students were required to attend a minimum of five sessions. Because of the relatively low rate of required attendance there was intentional redundancy within the curriculum in terms of the emphasized principles and learning points for each session. For instance, sessions on head trauma and cervical spine trauma were similar in that they both discussed the development and use of clinical decision tools.

During each of the first nine sessions, we reviewed two to three articles that were related by content. For example, the syncope session reviewed the original validation article of the San Francisco Syncope Rule and two subsequent external validation studies of the rule. Articles were chosen by the curriculum developers after review of the Academic Life in EM 2016 list of landmark articles.^7^ Articles were chosen to ensure the curriculum covered different topics within EM, reviewed different core topics within EBM such as research methodologies, and included the study of articles with conflicting results. This curriculum was certainly not designed to be comprehensive in its breadth of clinical or EBM topics. See Table 1 for a complete list of articles included within the curriculum.

Articles were distributed to the students via the course’s online learning platform, and they were expected to read the articles prior to each session. During the sessions, the articles were individually analyzed in a small- group discussion format, with one or two faculty members facilitating the discussion. Particular attention was paid to the clinical question, the research methodology, the results, the interpretation of the results, and the historical, clinical impact of the article, including its relation to other articles reviewed during that session.

The 10^th^ session was slightly different in format. It was attended by all the students taking part in the course, and they were split into two groups. Each group presented a critical analysis of a contemporary article and commented on whether and how it should change clinical practice. Although there was no formal evaluation of this presentation, it did allow students to independently apply the skills they had developed throughout the curriculum.

## IMPACT

Institutionally designed and mandated surveys assessing the quality of the sessions on a five-point Likert scale were distributed to the participating students after each session. A total of 15 students were enrolled in the curriculum and generated a total of 83 surveys. The overall rating of the sessions was positive with 79 (95.2%) scoring either a 4 or 5 on the Likert scale. Each survey contained two open-ended items: one asking for positive feedback, and the other asking for constructive feedback. Two authors conducted reflexive thematic review of these comments, which revealed three main themes: 1) students are supportive of the journal club model of teaching EBM; 2) students value discussion of study design and statistics; and 3) sessions should target key clinical topics.

The curriculum’s impact is limited both by our outcome measures and our cohort size. Although we can only present satisfaction data, the fact that this curriculum was the only formal EBM received by our students in the fourth year of medical school leads us to believe that its implementation was worthwhile. Our cohort size was limited by the number of students who are EM bound each year, but our cohort reflects the experience of students interested in EM at a large medical school; thus, our findings are likely applicable to institutions nationwide.

The implementation of this journal club-style curriculum designed to advance fourth-year medical students’ proficiency in EM Milestone 19 was positively reviewed by the target audience.

## Figures and Tables

**Table 1:** Journal club sessions and articles.

Session topic	Article citation
Stroke care	Brott T, Broderick, R, Kothari R, et al. Tissue Plasminogen Activator for Acute Ischemic Stroke. N Engl J Med. 1995;333(24):1581–8. Berkhemer OA, Fransen PS, Beumer D, et al. A Randomized Trial of Intraarterial Treatment for Acute Ischemic Stroke. N Engl J Med. 2015;372:11–20.
Head trauma	Kuppermann N, Holmes JF, Dayan PS, et al. Identification of children at very low risk of clinically-important brain injuries after head trauma: A prospective cohort study. Lancet Lond Engl. 2009;374(9696):1160–70. Stiell IG, Wells GA, Vandemheen K, et al. The Canadian CT Head Rule for patients with minor head injury. Lancet Lond Engl. 2001;357(9266):1391–6. Haydel MJ, Preston CA, Mills TJ, et al. Indications for computed tomography in patients with minor head injury. N Engl J Med. 2000;343(2):100–5.
Cervical spine trauma	Hoffman JR, Wolfson AB, Todd K, et al. Selective Cervical Spine Radiography in Blunt Trauma: Methodology of the National Emergency X-Radiography Utilization Study (NEXUS). Ann Emerg Med. 1998;32(4):461–9. Stiell IG, Wells GA, Vandemheen KL, et al. The Canadian C-Spine Rule for Radiography in Alert and Stable Trauma Patients. JAMA. 2001;286(15):1841–8.
Pulmonary embolism	Wells PS, Anderson DR, Rodger M, et al. Excluding pulmonary embolism at the bedside without diagnostic imaging: Management of patients with suspected pulmonary embolism presenting to the emergency department by using a simple clinical model and d-dimer. Ann Intern Med. 2001;135(2):98–107. Kline JA, Courtney DM, Kabrhel C, et al. Prospective multicenter evaluation of the pulmonary embolism rule-out criteria. J Thromb Haemost. 2008;6(5):772–80.
Cardiology treatments	Andersen HR, Nielsen TT, Rasmussen K, et al. A Comparison of Coronary Angioplasty with Fibrinolytic Therapy in Acute Myocardial Infarction. N Engl J Med. 2003;349(8):733–42. Wyse DG, Waldo AL, DiMarco JP, et al. A Comparison of Rate Control and Rhythm Control in Patients with Atrial Fibrillation. N Engl J Med. 2002;347(23):1825–33.
Syncope	Quinn J, McDermott D, Stiell I, et al. Prospective validation of the San Francisco Syncope Rule to predict patients with serious outcomes. Ann Emerg Med. 2006;47(5):448–54. Sun BC, Mangione CM, Merchant G, et al. External validation of the San Francisco Syncope Rule. Ann Emerg Med. 2007;49(4):420–427, 427.e1–4. Birnbaum A, Esses D, Bijur P, et al. Failure to validate the San Francisco Syncope Rule in an independent emergency department population. Ann Emerg Med. 2008;52(2):151–9.
Post cardiac arrest care	Holzer M, Cerchiari E, Martens P, et al. Mild Therapeutic Hypothermia to Improve the Neurologic Outcome after Cardiac Arrest. N Engl J Med. 2002;346(8):549–56. Nielsen N, Wetterslev J, Cronberg T, et al. Targeted Temperature Management at 33°C versus 36°C after Cardiac Arrest. N Engl J Med. 2013;369(23):2197–206.
Pediatrics	Bjornson CL, Klassen TP, Williamson J, et al. A Randomized Trial of a Single Dose of Oral Dexamethasone for Mild Croup. N Engl J Med. 2004;351(13):1306–13 Spiro DM, Tay K-Y, Arnold DH, et al. Wait-and-see prescription for the treatment of acute otitis media: A randomized controlled trial. JAMA. 2006;296(10):1235–41.
Sepsis	Rivers E, Nguyen B, Havstad S, et al. Early Goal-Directed Therapy in the Treatment of Severe Sepsis and Septic Shock. N Engl J Med. 2001;345(19):1368–77. Yealy MD, Kellum JA, Huang DT, et al. A Randomized Trial of Protocol-Based Care for Early Septic Shock. N Engl J Med. 2014; 370:1683–93.
Fluid resuscitation	Young P, Bailey M, Beasley R, et al. Effect of a Buffered Crystalloid Solution vs Saline on Acute Kidney Injury Among Patients in the Intensive Care Unit: The SPLIT Randomized Clinical Trial. JAMA. 2015;314(16):1701–10. Semler MW, Self WH, Wanderer JP, et al. Balanced Crystalloids versus Saline in Critically Ill Adults. N Engl J Med. 2018;378(9):829–39.
